# The Role of Governments in the Implementation of Patient Safety and Patient Safety Incident Reporting in Indonesia: A Qualitative Study

**DOI:** 10.3390/healthcare7020064

**Published:** 2019-04-24

**Authors:** Inge Dhamanti, Sandra G. Leggat, Simon Barraclough

**Affiliations:** 1 Department of Health Policy and Administration, Faculty of Public Health, Universitas Airlangga, Surabaya 60115, Indonesian; 2 Center for Patient Safety Research, Universitas Airlangga, Surabaya 60115, Indonesia; 3 School of Psychology and Public Health, La Trobe University, Victoria 3083, Australia; S.leggat@latrobe.edu.au (S.G.L.); s.barraclough@latrobe.edu.au (S.B.)

**Keywords:** patient safety, incident reporting, government roles, provincial health office, district health office

## Abstract

(1) Background: A patient safety incident reporting system was introduced in Indonesian hospitals in 2006; however, under-reporting of patient safety incidents is evident. The government plays a vital role in the implementation of a national system. Therefore, this study focuses on how the Indonesian government has been undertaking its role in patient safety at provincial and city/district levels, including incident reporting according to the National Guideline for Hospital Patient Safety. (2) Methods: This study employed a qualitative approach with interviews of 16 participants from seven organizations. The data were managed using NVivo and thematically analyzed. (3) Results: The findings revealed several problems at the macro-, meso-, and micro-level as the government was weak in monitoring and evaluation. The District Health Office (DHO) and Provincial Health Office (PHO) were not involved in incident reporting, and there was a lack of government support for the hospitals. (4) Conclusions: The DHO and PHO have not carried out their roles related to patient safety as mentioned in the national guidelines. Lack of commitment to and priority of patient safety, the complexity of the bureaucratic structure, and a lack of systematic partnership and collaboration are problems that need to be addressed by systematic improvement. To ensure effective and efficient national outcomes, the three levels of government need to work more closely.

## 1. Introduction

Patient safety incident reporting systems provide information on the occurrence of patient safety incidents to mitigate risk, improve the system, learn from the mistakes, and share learning [1,2,3,4]. Incident reporting in hospitals is usually governed by a national reporting system. In Indonesia, a patient safety incident reporting system was introduced in hospitals in 2006. The government has published supporting regulations and guidelines such as the National Guidelines for Hospital Patient Safety [5], the Guidelines for Patient Safety Incident Report [6], and other regulations [7,8]. Patient safety implementation, including incident reporting, was handled by the National Committee on Patient Safety, the Indonesian Hospital Association (IHA), and the Commission for Hospital Accreditation (CHA) [5] which is also responsible for hospital accreditation and delivering patient safety training. Government organizations, such as the Ministry of Health and District Health Offices (DHO), also carried out roles at the provincial and district/city levels, including advocacy for the patient safety program to the hospitals and advocacy for budget-related patient safety to the government [5] (please refer to Table 1).

Notwithstanding the efforts of all levels of government, the under-reporting of patient safety incidents is evident, as only around 668 incidents were reported in 2016 [9]. This number is very low compared to the number of accredited hospitals [10], which are supposed to report incidents to the National Committee. In addition, the yearly national patient safety incident report was not published because of confidentiality issues. At the hospital level, problems such as incomplete or unwritten reports, validation problems and adverse events not being followed up by investigation were found, and no effective feedback of learning or corrective actions were implemented to prevent the same incidents recurring [11,12].

Since the government plays a vital role in the implementation of a national system for improving patient safety, including tracking and monitoring patient safety incidents, this study focuses on how the Indonesian government has been undertaking its role in patient safety at the provincial and city/district levels. The focus of the study is on the implementation of patient safety incident reporting according to the National Guidelines for Hospital Patient Safety. 

## 2. Materials and Methods 

This study employed a qualitative approach using semi-structured interviews as the main data collection method. A qualitative method was chosen because it offers deeper understanding of the context and the processes that could not be addressed using a survey [13]. Semi-structured interview questions were developed and officials from government organizations and public hospitals from three cities or districts in East Java were interviewed. The data collected was managed using NVivo software prior to its thematic analysis. 

### 2.1. Study Design and Sample 

Semi-structured interviews were conducted with a purposeful sample of key informants from the government organizations expected to understand the issues regarding the implementation of patient safety incident reporting. In total, staff from seven organizations, comprising four government organizations and three public hospitals, were involved (please refer to Table 2). The three public hospitals were from three different districts and subject to the inclusion criteria that the hospital had been accredited in the last three years and the hospital was a referral center in the district or region. 

To ensure the most suitable informants were interviewed within each organization, the organizational chart was used to identify the informants who worked on patient safety or in the patient safety reporting area. For hospitals, the hospital director or manager, the head or secretary of the hospital patient safety team, and one head of the ward, were identified with the assumption that they understood the implementation of patient safety and patient safety incident reporting. Letters were then sent seeking approval to conduct a research study, requesting one or two organization representatives to be interviewed. Once approved, the people whose names had been given were contacted and the interviews were held at an agreed time and place. Seventeen participants were contacted at first, and only one from the District Health Office (DHO) from city B refused to participate, resulting in the involvement of 16 participants.

### 2.2. Data Collection and Analysis

The first author conducted the interviews in the Indonesian language, all of which were audio-recorded with the participant’s consent. The interviews lasted 30 minutes to one hour and took place at the participant’s offices. The recorded interviews were transcribed and then translated into English. Related themes were identified and the data were coded according to these themes. Nvivo software was used to manage the interview data. In preparing the manuscript, we followed the Consolidated Criteria for Reporting Qualitative Research (COREQ) checklist (please refer to supplementary materials, Table S1). 

## 3. Results

### 3.1. Roles Related to the Implementation of Patient Safety Initiatives

At the provincial level, the roles of Provincial Health Officer (PHO) were to serve as the regulator, to conduct the coaching programs and to require hospitals to obtain accreditation. Within the patient safety context, the participants from PHO emphasized that the PHO required all hospitals in the East Java region to be accredited and the accreditation itself constituted an effort to improve and manage patient safety and the quality of services. 

Regarding the roles mentioned in the guidelines, the PHO has undertaken advocacy for patient safety implementation by conducting meetings and inviting representatives from each hospital to attend. However, the PHO has not engaged in budget-related advocacy to the Provincial Government and District/City Government. As one participant explained:

Related to the budget…, in the last few years we have prioritized the achievement of the MDGs (Millennium Development Goals) whereas for the patient safety we motivated the hospitals to do [the patient safety program] with their own hospital funds [relying] neither upon the regional nor the state budget.

(JT2, provincial)

At the district and city levels, most participants agreed that government organization’s roles associated with patient safety were to conduct advocacy and coaching and to require hospitals to be accredited and issue hospital licenses. As described by one participant:

Our roles, according to the regulations, were focused on the safety of hospital buildings, licensing and hospital accreditation.

(SB1, District B)

Advocacy on the patient safety program was undertaken in the form of a meeting with hospitals every three months, although the agenda for these meetings was not restricted to patient safety. Another form of advocacy was the monitoring and evaluation of hospital services; this exercise was held every six months but was primarily concerned with hospital licensing, and therefore only indirectly related to patient safety. However, one participant highlighted that advocacy was only possible if the hospitals were under DHO supervision:

The DHO provided advocacy and coaching program[s] to hospitals under our supervision in the form of a three-monthly meeting.

(SD2, District A)

Regarding the advocacy on the budget, the participants reported:

We conducted the advocacy on patient safety program. However, we did not do any intervention related to budgeting.

(SB1, District B)

I did not have a special budget for patient safety programs. I included the patient safety [issue] in our quarterly meeting with hospital as the hospital coaching program.

(SD2, District A)

We have not advocated the policy maker to disburse funds or give more attention on certain regulations to reduce patient safety.

(GR2, District C)

Only one hospital implemented patient safety initiatives, such as hand-washing, socialization, and a band identification system, and did so with its own resources. “Socialization”, is an Indonesian term that refers to a process by which individuals acquire new knowledge on certain topics or a process by which people are exposed to appropriate ways of doing things. In this study, socialization is related to patient safety or incident reporting. The other two hospitals in the sample have not implemented patient safety activities. 

### 3.2. Roles Related to the Implementation of Incident Reporting

The implementation of patient safety incident reporting required internal reporting at the hospital level and external reporting directly from the hospitals to the National Committee, the national patient safety agency [3]. Incidents reported included near misses, adverse events and sentinel events. As of April 2019, there has been no comprehensive information about patient safety in Indonesia published on the National Committee website, nor did the Committee publish annual patient safety incident reports or share learning from reported incidents [14]. The National Committee did not provide any notification or feedback to the PHO or DHO regarding incident reporting in the local hospitals, and the hospital data went directly to the National Committee. As participants reported:

... the implementation of patient safety should ideally be reported to the Provincial Health Office, because the Health Office is responsible for health care in the region. But what has happened so far is that we have never received reports from hospitals.

(JT2, Provincial level)

Yes, indeed [we’re] not involved. The incidents are supposed to be reported to us, so we will know what has happened in this region.

(SD1, District A)

The condition left the PHO and DHO unaware of the patient safety incidents that had occurred in the hospitals in the region unless the patient reported the incident to the media or police. One head of DHO pointed out that he never received any socialization concerning patient safety in the district hospitals. Lack of direction from the Ministry of Health (MOH) regarding the implementation of the reporting system was also described:

The Ministry of Health has not provided a direction whether [the reporting has] entered into the mandatory program or not, and also the indicators related to the patient safety program do not exist yet.

(JT2, Provincial level)

The implementation of incident reporting in the sampled hospitals varied. Hospital A has implemented internal incident reporting but has not undertaken root cause analysis (RCA), Hospital B did not have incident reporting in place, while Hospital C had just started internal reporting. All hospital participants agreed that there were two main purposes for reporting patient safety incidents: to gather national data and to improve patient care. 

## 4. Discussion

This study revealed several problems at the macro-, meso- and micro-level for the implementation of patient safety programs and patient safety incident reporting in Indonesia. At the macro-level, the Indonesian government’s tasks are to develop the policy, set standards and undertake public assurance through a monitoring and reporting process [15]. However, monitoring and evaluation have not been a priority, as the performance of the roles listed in the reporting guidelines have never been evaluated. The patient safety policy development lacked adequate resources to implement the policy at all levels, which could have been detected and fixed earlier if the monitoring and evaluation functions were carried out at the national level. Despite its importance, the government lacked a coordinated mechanism to systematically assess, evaluate and follow up quality improvement measures [16]. This finding was strongly supported by the Republic of Indonesia Health System Review that highlighted the problems related to the implementation, as well as monitoring and evaluation of the impact of the regulations on quality and safety [17]. 

At the meso-level, despite the government’s efforts to increase attention to patient safety, the DHO and PHO have not carried out their roles related to patient safety detailed in the national guidelines, especially those requiring advocacy for patient safety programs to the hospital and patient safety budgeting. The advocacy program was carried out by the DHO and PHO by simply adding patient safety issues to existing programs or activities. Neither organization had a specific program or budget allocated for patient safety, which showed a lack of commitment to and priority of patient safety. 

Advocacy for patient safety programs on the part of the DHO and PHO did not extend to all hospitals in the region. This was primarily due to a complex bureaucratic structure that did not allow the DHO or PHO to work with all hospitals in the regions. This indicated lack of systematic partnership and collaboration in patient safety and lack of integration of disparate activities on patient safety [18]. These results support the need to establish a patient safety council and patient safety work-plans at the provincial and district levels, since the DHO and PHO do not have specific patient safety units [19]. Enhancing cooperative government and intergovernmental relations would also support the DHO and PHO in performing their patient safety roles [20]. 

The DHO and PHO have not been involved in patient safety incident reporting. This is in keeping with the national guidelines, since patient safety and incident reporting are managed by independent agencies without the involvement and coordination of the DHO and PHO. In Indonesia, lacked of transparency and openness in reporting and web-based dissemination of the reported incident data is in contrast with Taiwan and Malaysia, which have a patient safety dedicated website and systematic learning from incidents mechanisms in place [21,22]. This resulted in a missing link in the implementation of incident reporting between the national level and the provincial and district or city level, as the information about the incidents reported was concentrated at the national level and there was no feedback provided to the sub-national level. In organizing patient safety, it is crucial to engage different levels to work together to implement the program [23]. 

At the micro-level, the hospitals tried to allocate a budget for patient safety programs, however, this financial commitment varied among the hospitals. Some hospitals were able to fund their own programs, but others were not. According to the World Health Organization (WHO), the allocation of funds for patient safety programs and actions by the government demonstrate a commitment to improving the safety and quality of care in health care settings [24]. Political support and commitment from the government is critical to improving patient safety [25], however, this has not yet happened in Indonesia. 

Governments play a major role in achieving better quality with value in health care and in the success or failure of healthcare reform and improvement [26]. While regulation is formulated at the national level, implementation occurs mostly at the regional and district levels [27], thus monitoring and evaluation at every system level is needed to check whether the program or action is implemented as planned and has achieved the anticipated results [28]. This is essential to determine whether the program should be continued, modified or eliminated [29]. Monitoring and evaluation are complementary activities and need to be integrated with implementation, not as a distinct activity [20,28] and need to have clear key performance indicators. Within a local government context, carrying out monitoring and evaluation also demonstrates good governance and accountability [20]. However, these have not yet been carried out by the national level or the DHO and PHO in relation to patient safety. 

## 5. Conclusions

The Indonesian DHO and PHO have not optimally carried out their roles in implementing patient safety set out in the national guidelines. The lack of involvement of these organizations in managing incident reporting means that they are not aware of patient safety issues in the hospitals in their regions. Moreover, monitoring and evaluation were not undertaken. The lack of commitment to and priority of patient safety, the complexity of the bureaucratic structure, lack of systematic partnership and collaboration, and the lack of a framework to systematically assess, evaluate and follow up quality improvement measures are the problems that need to be addressed. The three levels of government need to work more closely by setting annual patient safety goals, performance indicators and performance agreements, and developing guidelines to be used by the regional or local level government organizations on how to achieve targets. Monitoring and providing feedback are also important for ensuring effective and efficient national outcomes. Lastly, all documents and the progress reports must be made publicly available as part of the organization’s accountability.

## Figures and Tables

**Table 1:** The government organizations and their related roles regarding patient safety implementation.

Organization	Level	Roles according to National Guidelines
National Government	National	Developing the policy Setting the standards Undertaking public assurance through a monitoring and reporting process
Provincial Health Office	Province	Conducting advocacy on the patient safety program to the hospitals in the region Conducting advocacy to the provincial government and district or city government for a budget to support the patient safety program in the hospitals Conducting coaching on the implementation of the patient safety program in the hospitals
District/City Health Office	District/city	Conducting advocacy on the patient safety program to the hospitals in the district or city area Conducting advocacy to the district/city level government for a budget to support the patient safety program in the hospitals Conducting coaching on the implementation of the patient safety program in the hospitals Encouraging the hospitals to achieve accreditation

**Table 2:** Number of interview participants and their organizations.

Organizations	Number of Participants
East Java Provincial Health Office	2
District A DHO	2
City B DHO	1
District C DHO	2
Public Hospital in District A	3
Public Hospital in City B	3
Public Hospital in District C	3
**Total**	**16**

## Supplementary Materials

The following are available online at https://www.mdpi.com/2227-9032/7/2/64/s1, Table S1: COREQ (Consolidated Criteria for Reporting Qualitative Research) Checklist.
