# Supplementary material for: The Role of Governments in the Implementation of Patient Safety and Patient Safety Incident Reporting in Indonesia: A Qualitative Study
Source: Healthcare (Basel). 2019 Apr 24;7(2):64. doi: 10.3390/healthcare7020064 (PMC6628004; doi:10.3390/healthcare7020064)
Supplement: Supplementary file 1 [file healthcare-07-00064-s001.pdf]

Supplemental Material

The Role of Governments in the Implementation of Patient Safety and Patient Safety Incident Reporting in Indonesia: A Qualitative Study

Inge Dhamanti, Sandra G. Leggat and Simon Barraclough

Table 1. COREQ (COnsolidated criteria for REporting Qualitative research) Checklist.

**Topic**	**Item No.**	**Guide Questions/Description**	**Reported on Page No.**
**Domain 1: Research team and reflexivity**			
Personal characteristics			
Interviewer/facilitator	1	Which author/s conducted the interview or focus group?	2.2. Data collection and analysis<br>The first author conducted the interviews in the Indonesian language, all of which were audio-recorded with the participant's consent.
Credentials	2	What were the researcher's credentials? E.g. PhD, MD	ID was a PhD student under SL and SB' supervision. ID is Indonesian female and the project was part of her doctoral thesis. She is a lecturer in Indonesia and had experiences in undertaking research. SL is a female professor in Health Services Management while SB is male Associate Professor with background in health policy and political economy of health systems in developing countries
Occupation	3	What was their occupation at the time of the study?	
Gender	4	Was the researcher male or female?	
Experience and training	5	What experience or training did the researcher have?	
Relationship with participants			

Relationship established	6	Was a relationship established prior to study commencement?	Not applicable. No prior relationship between the interviewers and interviewees existed.
Participant knowledge of the interviewer	7	What did the participants know about the researcher? e.g. personal goals, reasons for doing the research	ID sent an email to inform the potential participants about the study with the participant information statement approved by the research ethics committee attached. There was no previous personal connection between the researchers and participants.
Interviewer characteristics	8	What characteristics were reported about the inter viewer/facilitator? e.g. Bias, assumptions, reasons and interests in the research topic	ID as the interviewer has interests in patient safety.
**Domain 2: Study design**			
Theoretical framework			
Methodological orientation and Theory	9	What methodological orientation was stated to underpin the study? e.g. grounded theory, discourse analysis, ethnography, phenomenology, content analysis	**2. Materials and Methods**<br>This study employed a qualitative approach using the interview as the main data collection method.
Participant selection			
Sampling	10	How were participants selected? e.g. purposive, convenience, consecutive, snowball	2.1. Study design and sample<br>Semi-structured interviews were conducted with a purposeful sample of key informants from the government organizations expected to understand the issues regarding the implementation of patient safety incident reporting.
Method of approach	11	How were participants approached? e.g. face-to-face, telephone, mail, email	2.1. Study design and sample<br>Letters were then sent seeking approval to

			conduct a research study, requesting one or two organization representatives to be interviewed
Sample size	12	How many participants were in the study?	2.1. Study design and sample<br>Seventeen participants were contacted at first, and only one from the District Health Office (DHO) from city B refused to participate, resulting in the involvement of 16 participants
Non-participation	13	How many people refused to participate or dropped out? Reasons?	2.1. Study design and sample<br>Seventeen participants were contacted at first, and only one from the District Health Office (DHO) from city B refused to participate, resulting in the involvement of 16 participants.
Setting			
Setting of data collection	14	Where was the data collected? e.g. home, clinic, workplace	2.2. Data collection and analysis<br>The interviews lasted 30 minutes to one hour and took place at the participant's offices.
Presence of non-participants	15	Was anyone else present besides the participants and researchers?	NA
Description of sample	16	What are the important characteristics of the sample? e.g. demographic data, date	2.1. Study design and sample<br>To ensure the most suitable informants were interviewed within each organization, the organizational chart was used to identify the

			informants who worked on patient safety or in the patient safety reporting area.
Data collection			
Interview guide	17	Were questions, prompts, guides provided by the authors? Was it pilot tested?	**2. Materials and Methods**<br>We developed a semi-structured interview questions and interviewed the officials from government organisations and public hospital from three cities or districts in East Java.
Repeat interviews	18	Were repeat inter views carried out? If yes, how many?	NA
Audio/visual recording	19	Did the research use audio or visual recording to collect the data?	2.2. Data collection and analysis<br>The first author conducted the interviews in Indonesian language and all the interviews were audio-recorded with the participants' consent.
Field notes	20	Were field notes made during and/or after the interview or focus group?	NA
Duration	21	What was the duration of the inter views or focus group?	2.2. Data collection and analysis<br>The interviews lasted 30 minutes to one hour and took place at the participant's offices.
Data saturation	22	Was data saturation discussed?	Not Applicable, as this study this study used "qualitative description" so is no theory development process.
Transcripts returned	23	Were transcripts returned to participants for comment and/or	Transcripts were not returned to interviewees.
Domain 3: analysis and findings Data Analysis			
Data analysis			

Number of data coders	24	How many data coders coded the data?	ID was the primary data coder and the consistency was checked by the other two authors.
Description of the coding tree	25	Did authors provide a description of the coding tree?	2.2 Data collection and analysis<br>Related themes were identified and the data were coded according to these themes
Derivation of themes	26	Were themes identified in advance or derived from the data?	
Software	27	What software, if applicable, was used to manage the data?	2.2. Data collection and analysis<br>Nvivo software was used to manage the interview data
Participant checking	28	Did participants provide feedback on the findings?	We did not have any formal process of participant checking of research findings.
Reporting			
Quotations presented	29	Were participant quotations presented to illustrate the themes/findings? Was each quotation identified? e.g. participant number	Yes, in the result section.
Data and findings consistent	30	Was there consistency between the data presented and the findings?	We have attempted to present our findings in a clear and consistent manner.
Clarity of major themes	31	Were major themes clearly presented in the findings?	
Clarity of minor themes	32	Is there a description of diverse cases or discussion of minor themes?	
